# Recurrent aspiration pneumonia due to bilateral associated laryngeal paralysis after tooth extraction under general anesthesia: a case report

**DOI:** 10.1186/s40981-020-00335-6

**Published:** 2020-04-25

**Authors:** Kyotaro Koshika, Takashi Ouchi, Ryohei Serita, Toshiya Koitabashi

**Affiliations:** 1 Department of Anesthesiology, Tokyo Dental College Ichikawa General Hospital, 5-11-13 Sugano, Ichikawa City, Chiba, 272-8513 Japan; 2 Department of Intensive Care Medicine, National Research and Development Agency, National Cancer Center Hospital East, 6-5-1 Kashiwanoha, Kashiwa City, Chiba, 277-8577 Japan

**Keywords:** Associated laryngeal paralysis, Aspiration pneumonia, General anesthesia

## Abstract

**Background:**

Associated laryngeal paralysis is a clinical condition merged with other cranial nerve disorders associated with vocal cord paralysis. It is a rare complication in patients after general anesthesia. Here, we report our experience with a patient who developed associated laryngeal paralysis after oral surgery.

**Case presentation:**

A healthy 31-year-old man underwent extraction of horizontally impacted wisdom teeth in the bilateral mandible under general anesthesia. During the surgery, no significant changes in respiratory and cardiovascular parameters or neurosurgical abnormalities occurred. After the surgery, the patient was diagnosed with aspiration pneumonia. Furthermore, the results of otorhinolaryngological and neurological examinations led to a diagnosis of a combination of bilateral glossopharyngeal and vagus nerve paralysis, right recurrent nerve paralysis, and right hypoglossal nerve paralysis. In this case, seriously associated peripheral laryngeal paralysis with repeated episodes of aspiration pneumonia improved in approximately 6 months with rehabilitation and vitamin B12 administration, and no complications remained.

**Conclusions:**

We suggest that the anesthesiologist should take care of each procedure minutely. It is important to diagnose cases of nerve palsy as soon as possible to reduce the damage. Having had experience with this case, we believe sharing our experience with anesthesiologists is important.

## Background

Associated laryngeal paralysis is a clinical condition merged with other cranial nerve disorders associated with vocal cord paralysis [1] and is a rare complication in patients after general anesthesia [2]. In this study, we report our experience with a patient who developed postoperative associated laryngeal paralysis as well as a literature review. To the best of our knowledge, the combination of bilateral glossopharyngeal and vagus nerve, unilateral recurrent nerve, and unilateral hypoglossal nerve paralysis after transnasal intubation has not been previously reported.

## Case presentation

A 31-year-old man (180 cm, 87 kg) was admitted to our hospital for the extraction of horizontally impacted wisdom teeth in the bilateral mandible. His past history included no appreciable disease and no evidence of neck or head injuries. The preoperative neurological and anesthetic evaluation results were all within normal limits (American Society of Anesthesiologists physical status 1 and Mallampati score grade 1). Tooth extraction was conducted under general anesthesia. Anesthesia was induced by the intravenous administration of propofol and remifentanil, with neuromuscular blockade obtained with rocuronium. Transnasal intubation using a Macintosh laryngoscope (blade size 3, ACOMA Medical, Tokyo, Japan) was performed gently during the first attempt without any difficulty by a dental anesthesiologist with 6 years of experience in the field. A nasotracheal tube (*Portex*® *Ivory PVC* Soft Seal cuffed North Polar endotracheal tube; Smiths Medical Japan, Tokyo, Japan) with an internal diameter of 7.0 mm and an external diameter of 10.2 mm were secured at a depth of 29 cm in the left nasal cavity. No problem was encountered during intubation (Cormack grade 1). The cuff was inflated to a pressure of approximately 20 cm H_2_O, and the cuff pressure was continuously monitored and adjusted between 20 and 25 cm H_2_O during the surgery. General anesthesia was maintained with propofol and remifentanil. Mechanical ventilation was set as follows: a tidal volume of 800 ml and a respiratory rate of 10/min. With the use of a tongue depressor, a throat pack was inserted on the posterior part of the tongue to avoid the passage of blood into the respiratory tract. The surgery was carried out with the patient in a supine position with the neck slightly extended. During the surgery, the head and neck positions were changed from side to side several times, and no significant changes in respiratory and cardiovascular parameters or neurosurgical abnormalities occurred. At the end of the surgery, extubation of the nasotracheal tube was performed after the removal of the throat pack without any problems. Neuromuscular blockade was not reversed. The durations of the surgery and anesthesia were 221 min and 275 min, respectively. After a short observation in the operating room, the patient was transported to the ward.

After returning to the ward, the patient experienced hoarseness and difficulty swallowing. X-ray and computed tomography (CT) revealed an alveolar infiltrative shadow. In addition, an endoscopic evaluation showed unilateral vocal cord paralysis and aspiration, leading to a diagnosis of aspiration pneumonia. Treatment with intravenous sulbactam/ampicillin (SBT/ABPC) (6 g/day) was initiated immediately. Furthermore, since the tongue deviated towards the right upon protrusion, multiple cranial neuropathy was suspected, which led to a request for detailed otorhinolaryngological and neurological examinations. The results of the examinations led to a diagnosis of bilateral glossopharyngeal and vagus nerve paralysis, right recurrent nerve paralysis, and right hypoglossal nerve paralysis. The patient was not able to swallow at all. The patient underwent nonsurgical management with vitamin B_12_ (1500 mcg/day), together with speech and swallowing therapy. A nasogastric tube was placed to prevent aspiration and to supplement oral intake. The results of a meticulous neurologic examination of the other cranial nerves, including CT and magnetic resonance imaging (MRI), showed no evidence of the involvement of the central nervous system. Aspiration pneumonia gradually improved, with cycles of improvement and worsening. All cases of nerve paralysis improved in approximately 6 months, and no complications remained.

## Discussion

We encountered a case of serious associated peripheral laryngeal paralysis with repeated episodes of aspiration pneumonia after oral surgery. Although there are reports regarding the onset of associated laryngeal paralysis after oral surgery [2, 3], there are no reports on a combination of bilateral glossopharyngeal and vagus nerve paralysis, right recurrent nerve paralysis, and right hypoglossal nerve paralysis after tooth extraction under general anesthesia so far. In our case, MRI images and the healing process ruled out central nerve origin complications so that peripheral neuropathy was possibly considered.

Peripheral neuropathy after general anesthesia may be caused by factors associated with anesthesia, body position, or surgery. Mask ventilation [4], laryngoscopy [5, 6], and the tracheal tube or cuff have been reported as factors associated with anesthesia [7, 8]. In our case, mask ventilation, visualization of the larynx, and nasotracheal intubation were all performed easily, so they were thus unlikely to cause neuropathy. In addition, the cuff pressure was continuously monitored, and no nitrous oxide was given in our case. In oral surgeries under general anesthesia, there are many factors that can induce neuropathy, such as frequent changes in the head and neck position, vocal cord tissue compression by tracheal tubes, and too much pressure of the tracheal cuff. In our case, the surgeon moved the patient’s neck from side to side several times during the surgery. However, there was no abnormality in the position of the patient’s neck or tracheal tube in our view, and no abnormal changes were observed in the cuff pressure. Direct nerve injury due to surgery, compression due to excessive pharyngeal packing [3], and compression from the use of a tongue depressor can be considered surgical factors. In our case, due to the performance of routine surgical procedures for tooth extraction and the anatomical distance between the surgical field and the paralyzed nerve, surgical factors could not serve as the cause of neuropathy. In particular, the fact that soft palate paralysis developed bilaterally made it difficult to identify the cause. Since the pharyngeal plexus provides the motor and sensory branches to the soft palate and pharynx [9], it might have been impaired due to some cause during surgery. Although there was no clear mechanism of injury for the nerves in this patient, several of the causes described above may have been combined.

As described above, the cause of associated laryngeal paralysis has not yet been identified in our case. However, this case demonstrated that seriously associated peripheral laryngeal paralysis with repeated episodes of aspiration pneumonia after oral surgery could improve in approximately 6 months with rehabilitation, including speech and swallowing therapy and vitamin B12 administration. Although associated laryngeal paralysis varies to some extent in terms of the time to healing, the time to healing of 6 months was nearly average [10]. In the present case, the onset of aspiration pneumonia led to the detection of associated laryngeal paralysis. It is important that associated laryngeal paralysis be considered a very rare complication after oral surgery under general anesthesia and be treated promptly in the case of disease onset.

## Conclusions

It is not so rare that each of the nerves including the glossopharyngeal nerve, vagus nerve, and hypoglossal nerve are paralyzed after surgery. However, there is no report on the occurrence of these three nerves being paralyzed simultaneously. We suggest that the anesthesiologist should take care of each procedure minutely, including mask ventilation, tracheal intubation, and close monitoring of cuff pressure. It is important to diagnose cases of nerve palsy as soon as possible to reduce the damage. Having had experience with this case, we believe that sharing our experience with anesthesiologists is important.

## Data Availability

Not applicable

## Abbreviations

CRP: C-reactive protein
WBC: White blood cell
CT: Computed tomography
SBT/ABPC: Sulbactam/ampicillin
MRI: Magnetic resonance imaging
